# Comparative study of ocular and generalized myasthenia gravis in a South Korean cohort

**DOI:** 10.1371/journal.pone.0346981

**Published:** 2026-04-28

**Authors:** Chaerin Kwon, Jeeyoung Oh, Hyun Jin Shin

**Affiliations:** 1 Graduate School of Medicine, Konkuk University, Seoul, Republic of Korea; 2 Department of Neurology, Konkuk University Medical Center, Seoul, Republic of Korea; 3 Research Institute of Medical Science, Konkuk University School of Medicine, Seoul, Republic of Korea; 4 Department of Ophthalmology, Konkuk University Medical Center, Seoul, Republic of Korea; Shuguang Hospital Affiliated to Shanghai University of Traditional Chinese Medicine, CHINA

## Abstract

**Purpose:**

To compare ocular myasthenia gravis (OMG) and generalized myasthenia gravis (GMG) in a South Korean cohort through an integrated analysis of clinical characteristics, diagnostic findings, and treatment patterns.

**Methods:**

We conducted a retrospective study of 138 patients with myasthenia gravis (98 with OMG and 40 with GMG). Demographic characteristics, ophthalmic symptoms, serologic results, repetitive nerve stimulation test (RNST) findings, thymic pathology, autoimmune comorbidities, and treatment patterns were evaluated.

**Results:**

GMG patients had a younger age at symptom onset and a higher proportion of females compared with OMG patients. Combined horizontal and vertical diplopia was more frequent in OMG. AChR antibody positivity was significantly higher in GMG than in OMG (92.5% vs. 36.3%), as were abnormal RNST findings (90.0% vs. 23.5%). A modest but statistically significant positive correlation was observed between AChR-Ab titer and the highest CMAP decrement in the overall cohort (r = 0.381, p = 0.011); however, this association was not significant when analyzed separately in OMG and GMG patients. Thymic abnormalities, particularly thymoma, were more common in GMG. Nonsteroidal immunosuppressant use—especially tacrolimus and azathioprine—was significantly more frequent in GMG, whereas corticosteroid use did not differ significantly between subtypes. Among OMG patients, systemic immunotherapy was more frequently administered to those presenting with both ptosis and diplopia than to those with isolated ptosis.

**Conclusions:**

In this South Korean cohort, OMG and GMG exhibited distinct demographic, serologic, electrophysiologic, and therapeutic profiles. Recognition of these subtype-specific differences may facilitate accurate diagnosis, individualized treatment selection, and improved clinical management.

## Introduction

Myasthenia gravis (MG) is a B-cell-mediated autoimmune disease characterized by muscle weakness and fatigability, and is primarily caused by autoantibodies targeting the acetylcholine receptor (AChR) at the neuromuscular junction [1]. MG can be classified into two main forms: ocular MG (OMG) and generalized MG (GMG). OMG is characterized by symptoms restricted to the eye muscles, primarily manifesting as ptosis and diplopia [2]. In contrast, GMG affects muscles beyond the eyes, potentially involving weakness in facial, neck, limb, and respiratory muscles [3,4] Although ocular myasthenia gravis is initially confined to the ocular muscles, longitudinal studies consistently indicate that secondary generalization is common. Population-based and long-term cohort data suggest generalization rates of approximately 30–55% [5,6], and a recent systematic review reported a pooled estimate of 39%, with considerable heterogeneity across studies related to follow-up duration and patient selection [7]. This wide variability is influenced by factors such as AChR antibody (AChR-Ab) positivity, abnormal findings in electrophysiologic testing, presence of thymic abnormalities, and the use or timing of immunosuppressive therapy.

Comparing OMG and GMG is clinically meaningful, as these subtypes differ in disease distribution, severity, and treatment strategies. Early identification of patients with OMG who are at high risk of progressing to GMG allows for timely interventions, and early immunotherapy has been shown to reduce the risk of generalization and improve long-term outcomes. Although the distinction between the two forms is widely recognized, previous studies have reported variable findings across populations regarding serologic markers, thymic pathology, and demographic patterns.

For example, one Western study found several similarities between OMG and GMG in Caucasian patients [8]: the rates of AChR-Ab positivity were similarly high (94.5% in OMG vs. 95.9% in GMG), and no significant differences were observed in thymic pathology, sex distribution, or the prevalence of other autoimmune diseases. These findings suggest minimal clinical distinction between the two subtypes in that population. However, data from East Asian cohorts remain limited, despite known racial differences in immune profiles and treatment responses [9,10].

To address this gap, the present study aimed to characterize the clinical, serologic, and electrophysiologic differences between OMG and GMG in a South Korean cohort by examining demographics, ophthalmic symptoms, antibody status, repetitive nerve stimulation test (RNST), findings, thymic pathology, autoimmune comorbidities, and treatment strategies. The goal was to support the development of individualized diagnostic and therapeutic approaches based on subtype-specific profiles.

## Materials and methods

This retrospective comparative cohort study was conducted at a single tertiary referral center (Konkuk University Medical Center, Seoul, South Korea), which operates a neuro-ophthalmology clinic specializing in the diagnosis and management of MG and other neuro-ophthalmic disorders, between August 2005 and December 2023. This study was conducted in accordance with the principles of the Declaration of Helsinki. The study protocol was reviewed by the Institutional Review Board (IRB) of Konkuk University Medical Center (Approval No. KUMC IRB 2025-03-011) and was determined to be exempt from the requirement for informed consent. As this was a retrospective study, patient data were anonymized prior to analysis. The data used in this study were accessed for research purposes between March 18, 2025, and June 18, 2025.

The diagnosis of MG was based on characteristic clinical symptoms—such as fluctuating muscle weakness, particularly involving the ocular muscles (ptosis and diplopia), and/or bulbar, facial, cervical, respiratory, or limb muscles—along with at least one confirmatory test, including AChR-Ab positivity, abnormal repetitive nerve stimulation (RNST) findings, or a significant response to pyridostigmine. Diagnostic decisions were made by consensus between an ophthalmologist (H.J.S.) and a neurologist (J.Y.O.). Patients were included if they were 18 years of age or older and diagnosed with MG according to these criteria [ 11,12]. The exclusion criteria included (1) insufficient medical records at the time of diagnosis, (2) uncertain symptom classification at onset, (3) coexisting neurologic or ophthalmologic diseases that could interfere with an MG diagnosis (e.g., thyroid-associated ophthalmopathy, strabismus, chronic progressive external ophthalmoplegia, or cranial nerve palsy), or (4) drug-induced MG.

The RNST was applied to the orbicularis oculi, trapezius, and abductor digiti minimi muscles. The compound muscle action potential (CMAP)—a summated electrical response recorded from a muscle when multiple motor nerve fibers are stimulated simultaneously—was measured for each muscle. The CMAP decrement was calculated as the percentage reduction between the first CMAP amplitude and the lowest of the fourth or fifth CMAP amplitude. The largest decrement was recorded for each muscle, and patients were classified as RNST abnormality if the decrement exceeded 10% in any tested muscle [11]. Additionally, all patients underwent contrast-enhanced CT of the chest to screen for thymic abnormalities such as thymoma and thymic hyperplasia.

Patients were classified as having OMG or GMG according to the Myasthenia Gravis Foundation of America (MGFA) clinical classification system, which categorizes the disease into five distinct classes based on the severity and distribution of muscle weakness [13]. OMG was defined as the presence of ptosis, diplopia, or both, with symptoms limited to the ocular muscles (MGFA Class I). For this study, patients were classified as OMG only if no bulbar, limb, respiratory, or axial muscle weakness developed throughout the documented follow-up period. Patients who initially presented with ocular symptoms but subsequently progressed to generalized weakness during follow-up were reclassified as GMG and were not retained in the OMG group. GMG was defined as the development of weakness involving bulbar, facial (excluding orbicularis oculi), cervical, respiratory, or limb muscles (MGFA Classes II to V). Classes II, III, and IV represent mild, moderate, and severe generalized weakness, respectively, each further subdivided into ‘a’ (limb and axial muscle involvement) and ‘b’ (bulbar and respiratory involvement). Class V denotes the most severe form of GMG, characterized by profound generalized weakness requiring intubation.

### Outcome measures

Outcome measures were designed for evaluating differences between OMG and GMG across five domains: (1) baseline clinical and demographic characteristics, including age at symptom onset and sex distribution; (2) ophthalmic manifestations, specifically the presence and patterns of ptosis and diplopia; (3) serological and electrophysiologic findings, including AChR antibody status and results of RNST, with emphasis on CMAP decrement and the muscle exhibiting the maximal decrement; (4) associated conditions, including thymic pathology (thymoma or thymic hyperplasia) and coexisting autoimmune diseases; and (5) treatment-related variables, including the use and duration of immunosuppressive therapy.

### Statistical analyses

All statistical analyses were performed using SPSS (version 27.0, IBM Corporation, Chicago, IL, USA). Continuous variables are presented as mean ± standard deviation when normally distributed and as median (interquartile range) when not normally distributed. Categorical variables are expressed as absolute numbers and percentages. Normality of continuous variables was assessed using the Shapiro–Wilk test. For comparisons between patients with OMG and GMG, continuous variables with normal distribution were analyzed using the Student’s t-test, whereas non-normally distributed variables were compared using the Mann–Whitney U test. Categorical variables were compared using Pearson’s chi-square test when the expected cell count was ≥ 5; Fisher’s exact test was applied when expected cell counts were <5. The relationship between AChR antibody concentration and highest CMAP decrement values was assessed using Spearman’s rank correlation analysis. P -value < 0.05 was considered statistically significant.

## Results

### Baseline clinical and demographic characteristics

The study included 138 patients diagnosed with MG, comprising 98 with OMG and 40 with GMG, with a median follow-up of 75.0 (45.0–129.75) months (range: 24–216 months) (Table 1). The overall female-to-male ratio was 1.34:1, with the proportion of males being significantly higher in the OMG group than in the GMG group (*p* = 0.026), and with a higher prevalence of GMG in females. The age at diagnosis was 49.44 ± 16.51 years, and was significantly lower in the GMG group than in the OMG group (*p* = 0.041). The mean age at symptom onset was significantly lower in the GMG group than in the OMG group (43.11 ± 16.38 vs 51.37 ± 16.14 years, *p* = 0.049).

**Table 1:** Baseline clinical and demographic characteristics of patients with ocular myasthenia gravis (OMG) and generalized myasthenia gravis (GMG).

	All MG (n = 138)	OMG (n = 98)	GMG (n = 40)	p
Sex, male	59 (42.7)	47 (47.9)	12 (30.0)	0.026^*^
Age, years	49.44 ± 16.51	51.21 ± 16.21	45.12 ± 16.53	0.041^†^
Age at symptom onset, years	48.98 ± 16.64	51.37 ± 16.14	43.11 ± 16.38	0.049^†^
Follow-up duration	75.0 (45.0–129.75)	85 (48.0–137.0)	70.0 (47.0–122.25)	0.032^‡^
Males	49.23 ± 16.98	50.91 ± 18.14	46.86 ± 13.58	
Females	47.71 ± 17.21	51.78 ± 17.34	41.72 ± 18.03	
Thymectomy	33 (23.9)	9 (9.1)	24 (57.5)	<0.001^*^
Duration from symptom onset to thymectomy, months	4.2 (1.0–20.0) (n = 33/36)	8.1 (4.0–24.25) (n = 9/10)	2.1 (1.0–16.25) (n = 24/26)	0.115^‡^
AChR-Ab positivity	74/138 (53.6)	37/98 (36.3)	37/40 (92.5)	<0.001^*^
Positive pyridostigmine test^††^	44/54 (81.5)	30/38 (78.9)	14/16 (87.5)	0.705^§^
Abnormal RNST	59/138 (42.8)	23/98 (23.5)	36/40 (90.0)	<0.001^*^

Data are presented as mean ± standard deviation, median (interquartile range), or number (%), as appropriate.

Follow-up duration and duration from symptom onset to thymectomy are expressed as median (interquartile range). For thymectomy-related variables, “n = available data/ total eligible patients” indicates the number of patients with available data.

^††^Percentages were calculated based on the number of patients with available data when applicable.

AChR-Ab, acetylcholine receptor antibody; RNST, repetitive nerve stimulation test.

* Pearson’s chi-square test

^†^Student’s t-test

^‡^Mann–Whitney U test

^§^Fisher’s exact test

### Ophthalmic manifestation

Ophthalmic symptoms (Table 2) were common in both groups. Fluctuating ptosis was observed in 120 of 138 patients (86.9%), and diplopia observed in 102 of 138 patients (73.9%). Among the 98 OMG patients, 27 (27.6%) had ptosis only, 11 (11.2%) had diplopia only, and 60 (61.2%) had both symptoms. Among patients who experienced diplopia, combined horizontal and vertical diplopia was significantly more frequent in the OMG group than in the GMG group (33.8% vs. 12.9%, p = 0.047).

**Table 2:** Comparison of ophthalmic manifestations between OMG and GMG patients.

	All MG (n = 138)	OMG (n = 98)	GMG (n = 40)	p
Ptosis only	36 (26.1)	27 (27.6)	9 (22.5)	0.549^†^
Diplopia only	18 (13.0)	11 (11.2)	7 (17.5)	0.320^†^
Both ptosis and diplopia	84 (60.9)	60 (61.2)	24 (60.0)	0.641^†^
Type of diplopia				
Horizontal only	23 (22.5)	17 (23.9)	6 (19.4)	0.484^†^
Vertical only	20 (19.6)	13 (18.3)	7 (22.6)	0.521^†^
Both horizontal and vertical	28 (27.5)	24 (33.8)	4 (12.9)	0.047^†^
Indeterminate^*^	31 (30.4)	17 (23.9)	14 (45.2)	0.013^†^
Ocular discomfort	16 (11.6)	14 (14.3)	2 (5.0)	0.095^‡^
Blurred vision	28 (20.2)	22 (21.4)	6 (12.5)	0.207^†^
Tearing	12 (8.7)	9 (9.2)	3 (7.5)	>0.999^‡^
Dry eye	18 (13.0)	13 (13.2)	5 (12.5)	0.918^†^
Photophobia	4 (2.9)	2 (2.0)	2 (5.0)	0.584^‡^
Cogan’s lid twitch	43 (31.2)	34 (34.7)	9 (22.5)	0.200^†^

Data are number (percentage) values.

Percentages for diplopia subtypes were calculated among patients with diplopia (diplopia only + both ptosis and diplopia).

*Fluctuating binocular diplopia that could not be reliably categorized.

†Pearson’s chi-square test; ^‡^, Fisher’s exact test.

### Serological and electrophysiologic findings

The MGFA classification differed significantly with the AChR-Ab status and RNST findings (Table 3). Patients with AChR-Ab positivity were significantly more likely to present with GMG (MGFA classes II–V), while those who were seronegative were more likely to have OMG (MGFA class I); this difference was highly statistically significant (*p* < 0.001). Similarly, abnormal RNST findings were strongly associated with higher MGFA classifications (particularly classes II–V), while normal RNST findings were more common in patients with MGFA class I (*p* < 0.001).

**Table 3:** Association of MGFA clinical class with serologic and electrophysiologic findings.

MGFA class at disease onset	All	AChR-Ab positive (n = 74/138)	AChR-Ab negative (n = 64/138)	Abnormal RNST (n = 59/138)	Normal RNST (n = 79/138)
I	98 (71.0)	37 (50.0)	61 (95.3)	23 (39.0)	75 (94.9)
II	28 (20.3)	26 (35.1)	2 (3.0)	25 (42.4)	3 (3.8)
III	6 (4.3)	5 (6.8)	1 (2.0)	5 (8.5)	1 (1.3)
IV	3 (2.2)	3 (4.1)	0 (0.0)	3 (5.1)	0 (0.0)
V	3 (2.2)	3 (4.1)	0 (0.0)	3 (5.1)	0 (0.0)
P		<0.001^†^		<0.001^†^	

Data are number (percentage) values.

MGFA, Myasthenia Gravis Foundation of America. ^†,^ Fisher’s exact test

**Fig 1:**
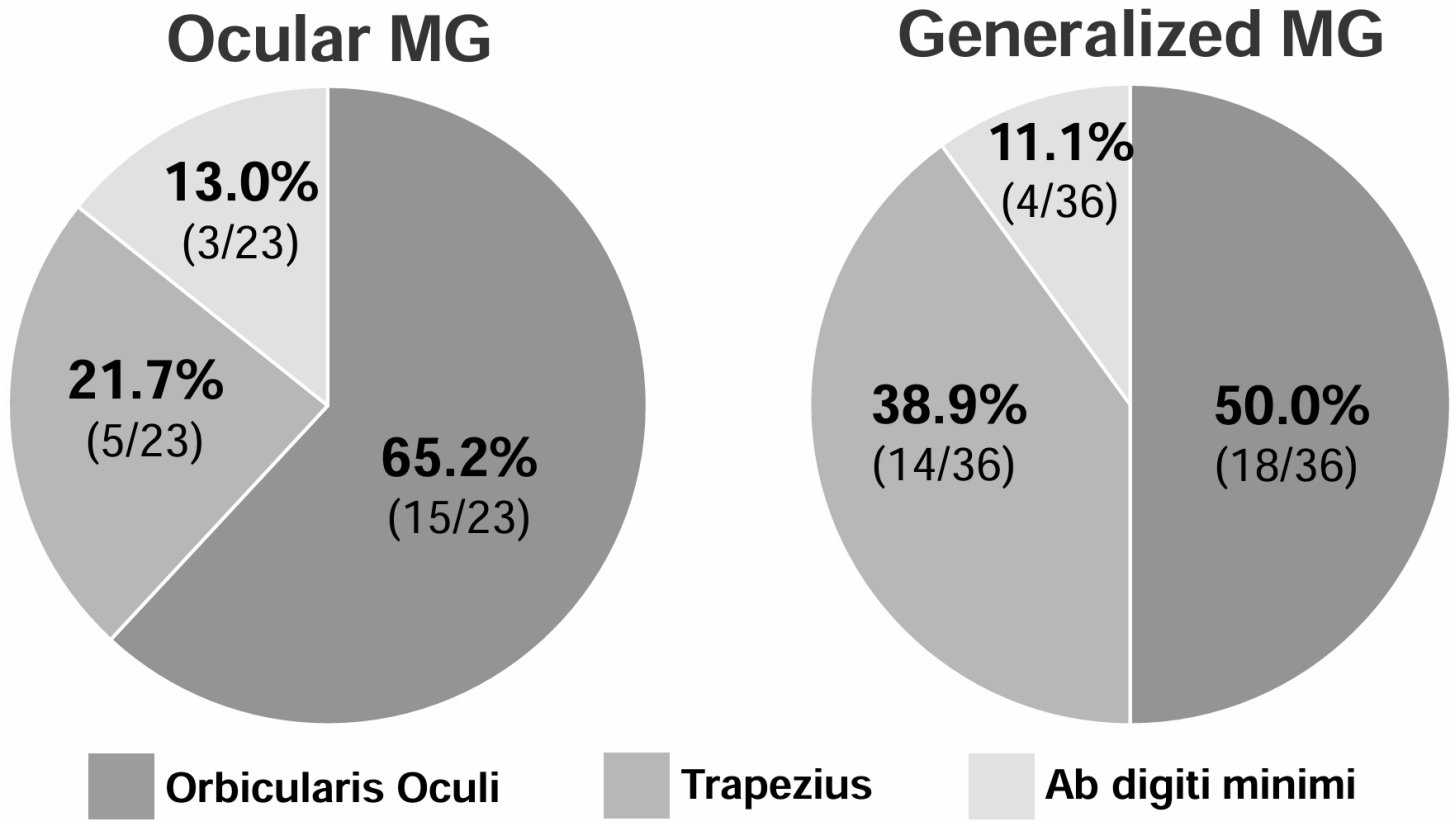
Frequency of muscles with the highest CMAP decrement. The orbicularis oculi muscle was the most affected muscle on RNST in both OMG and GMG patients.

Among patients with abnormal RNST findings (23 with OMG and 36 with GMG), the orbicularis oculi was the most frequently involved muscle in both the OMG and GMG groups (65.2% and 50.0%, respectively) (Fig 1). Overall, the proportion of patients with RNST abnormalities was significantly higher in the GMG group than in the OMG group (*p* < 0.001) (Table 1). However, when comparing the muscle that showed the largest CMAP decrement in each patient (i.e., the muscle with the largest percentage drop in CMAP amplitude among those tested), no significant difference was found between the two groups (*p* = 0.176). This suggests that the overall qualitative pattern of muscle involvement in the RNST did not differ between OMG and GMG.

**Fig 2:**
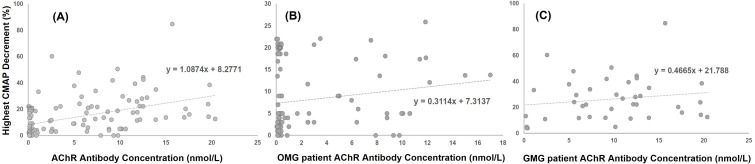
RNST-antibody correlation. A: There is a positive correlation of highest CMAP decrement with the concentration of AChR antibodies in the overall cohort (*r* = 0.381, *p* = 0.011). B, C: There is no clear correlation of highest CMAP decrement with the concentration of AChR antibodies in both OMG and GMG patients (B: *r* = 0.280, *p* = 0.068, C: *r* = –0.062, *p* = 0.671).

**Fig 3:**
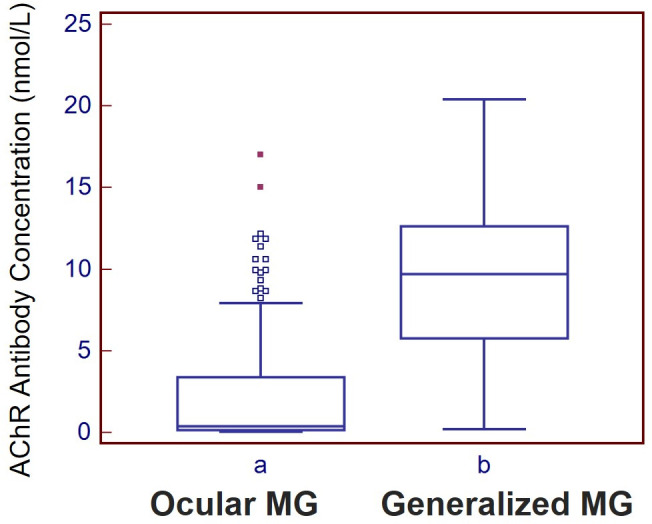
Distribution of AChR antibody concentrations in ocular and generalized myasthenia gravis. Box-and-whisker plots comparing AChR antibody concentrations between OMG and GMG. The median is shown by the horizontal line within each box, the box indicates the interquartile range, the whiskers represent the non-outlier range, and the points denote outliers. GMG showed higher antibody concentrations and a broader distribution than OMG.

The mean AChR-Ab titer was 2.69 nmol/L in OMG and 9.71 nmol/L in GMG (normal <0.5 nmol/L) (p < 0.001). In the overall cohort (Fig 2A), a positive correlation was observed between AChR-Ab level and highest CMAP decrement (r = 0.381, p = 0.011). When analyzed by disease subtype, this association did not reach statistical significance in patients with OMG (Fig 2B; r = 0.173, p = 0.087) and was absent in patients with GMG (Fig 2C; r = 0.160, p = 0.323). In OMG patients, highest CMAP decrement values were generally lower, with most values clustered below 25%. In contrast, GMG patients demonstrated a broader distribution of highest CMAP decrement values, indicating greater heterogeneity in electrophysiologic findings (Fig 3). These results suggest that while AChR-Ab levels may show a limited association with electrophysiologic measures in the overall cohort, they do not reliably reflect electrophysiologic severity within disease subtypes.

The pyridostigmine test demonstrated consistently high positivity in both groups, with no significant difference between OMG and GMG (*p* = 0.705), implying a robust pharmacologic responsiveness regardless of the disease subtype (Table 1).

### Associated conditions

Thymic abnormalities were significantly more common in patients with GMG than in those with OMG. Thymoma was identified in 23 patients overall, including 16 with GMG (40.0%) and 7 with OMG (7.1%) (*p* < 0.001). Thymic hyperplasia was observed in 19 patients—10 with GMG (25.0%) and 9 with OMG (9.2%)—and was also significantly more prevalent in GMG (*p* = 0.016). Thymectomy was performed in 33 patients overall (23.9%), including 24 with GMG (60.0%) and 9 with OMG (9.2%), showing a significantly higher frequency in GMG patients (*p* < 0.001). Thymoma was diagnosed radiologically and confirmed by histopathology following surgery in all applicable cases. Regarding autoimmune comorbidities such as hyperthyroidism and rheumatoid arthritis were observed in both OMG (15.3%) and GMG (12.5%), but their prevalence did not differ significantly between the two groups (*p* = 0.451) (S1 Table).

### Treatment-related variables

The treatment regimens primarily included pyridostigmine, corticosteroids, and immunosuppressants. Corticosteroid use was comparable between the two groups, with 78.0% of OMG patients and 97.5% of GMG patients receiving steroids (*p* = 0.067). In contrast, nonsteroidal immunosuppressants were prescribed significantly more often in GMG patients (75%) than in OMG patients (35.7%) (*p* < 0.001). Specifically, tacrolimus and azathioprine were significantly more common in GMG than in OMG patients. Mycophenolic acid and cyclosporine were used less frequently, and their usage rates did not differ significantly between the two groups (Table 4). These findings highlight that the immunosuppressive approach is typically more aggressive in GMG than in OMG. Among patients with ocular symptoms, those presenting with both ptosis and diplopia were more likely to receive corticosteroids or immunosuppressants than those with ptosis alone (*p* = 0.003), suggesting that a greater disease burden influenced treatment intensity.

**Table 4:** Use of non-steroidal immunosuppressive agents among patients with OMG and GMG.

	All MG (n = 138)	OMG (n = 98)	GMG (n = 40)	p
Any Immunosuppressant use	65 (47.1)	35 (35.7)	30 (75)	<0.001^*^
Azathioprine	40 (29.0)	22 (22.4)	18 (45.0)	0.040^*^
Mycophenolic acid	19 (13.8)	11 (11.2)	8 (20.0)	0.172^*^
Tacrolimus	37 (26.8)	13 (13.3)	24 (60.0)	<0.001^*^
Cyclosporine	4 (2.9)	2 (2.0)	2 (5.0)	0.581^†^

Data are presented as number (percentage) of patients. Percentages are calculated based on the total number of patients in each group.

Each agent was counted independently. Because some patients received more than one non-steroidal immunosuppressive agent and others received none, percentages do not sum to 100%.

*Pearson’s chi-square test

^†^Fisher’s exact test

## Discussion

Although each component of MG—such as serologic status, electrophysiologic findings, thymic pathology, and treatment patterns—has been previously studied, an integrated analysis of these factors within a well-characterized East Asian cohort has been lacking. Ocular and generalized myasthenia gravis represent clinically distinct phenotypes with meaningful differences in demographic profiles, diagnostic findings, and treatment requirements. In this South Korean cohort, generalized MG was more frequently associated with younger age at onset, female predominance, higher rates of AChR antibody positivity and RNST abnormalities, thymic pathology, and the need for systemic immunosuppressive therapy. In contrast, ocular MG tended to present with localized ophthalmic manifestations, including multidirectional diplopia, and generally required less intensive treatment.

Among the 138 patients in this study, females predominated in both the OMG and GMG groups, with a significantly higher proportion in the latter. This pattern aligns with previous reports on East Asian populations [14], but differs from Western data indicating a male predominance in OMG and no sex difference in GMG [5]. Delayed OMG diagnoses were more common than delayed GMG diagnoses, which might be attributable not only to the milder and more-localized symptoms in OMG, but also to underrecognition by clinicians. Because early-stage OMG can be subtle and easily misdiagnosed, clinicians should maintain a high index of suspicion for MG, especially in patients presenting with both horizontal and vertical diplopia.

Ptosis and diplopia were the most common initial symptoms in both OMG and GMG, but a subset of GMG patients presented without ocular involvement, suggesting that while ophthalmic signs are typical, they are not essential for making a definitive diagnosis. This finding is consistent with a Thai study finding that OMG patients more often presented with both ptosis and diplopia than did those who progressed to GMG [15]. Such symptom combinations may reflect a distinct clinical trajectory, although their predictive value remains uncertain. While an ophthalmic evaluation is essential in MG, specific ocular features are of limited value in distinguishing between subtypes or in forecasting disease generalization.

In this study, the pyridostigmine test showed high sensitivity in both GMG (87.5%) and OMG (78.9%), supporting its utility as an initial diagnostic tool in East Asian patients. Although there have been few direct comparisons, these values are broadly consistent with the sensitivity of neostigmine and edrophonium tests reported in Caucasian populations, which range from 84% in GMG and 60% in OMG [16]. Given its accessibility and good performance, pyridostigmine remains a practical and effective option for the initial screening of MG, though it does not reliably differentiate between OMG and GMG.

Serologic and electrophysiologic tests have demonstrated more-pronounced differences by subtype. In our cohort, AChR-Ab positivity was present in 92.5% of GMG patients and 36.3% of OMG patients. These findings align with reported Western data (85% in GMG, 50% in OMG) [17], South Indian data (80–90% in GMG, ~ 50% in OMG) [18], and another South Korean study showing that the positivity rate was 1.7-fold higher in GMG than in OMG [1]. Similarly, the sensitivity of the RNST was 90% in GMG and 23.5% in OMG in our study, comparable to values reported for Caucasian populations (80–89% in GMG, 54–62% in OMG) [14,19]. These consistent cross-regional patterns reinforce the diagnostic value of the AChR-Ab titer and the RNST in identifying generalized disease, while also illustrating the lower utility of these tests in patients with ocular-limited disease.

A statistically significant correlation between AChR-Ab titer and highest CMAP decrement was observed in the overall cohort, consistent with previous Western studies reporting only minimal direct associations between these parameters [20]. When analyzed by disease subtype, this association did not reach statistical significance in OMG patients and was absent in GMG patients, indicating that AChR-Ab titer does not reliably reflect electrophysiologic severity at the subtype level. These findings suggest that while AChR-Ab levels may show a limited association with electrophysiologic measures in aggregated analyses, their utility as markers of disease severity is restricted, particularly in generalized disease. This interpretation is further supported by prior observations that GMG patients may exhibit substantial RNST abnormalities even when clinical symptoms are mild or well controlled [8].

RNST abnormalities were markedly more frequent in GMG than in OMG, consistent with previous reports demonstrating higher diagnostic sensitivity of RNST in GMG compared with ocular-limited disease [14,19]. Regarding muscle involvement, the orbicularis oculi was the most commonly affected muscle in both OMG and GMG, and no meaningful difference in the qualitative pattern of muscle involvement was observed between the two groups, contrasting with some Western studies that reported a more evenly distributed pattern in GMG [8]. In addition, seronegative patients more frequently exhibited lower MGFA classifications, in line with prior reports suggesting that seronegative myasthenia gravis tends to remain ocular-limited [21], although other studies using alternative classification systems have reported no significant differences by serostatus [6]. While RNST abnormalities were generally associated with higher MGFA classes at the group level, the degree of CMAP decrement did not consistently correlate with clinical severity in individual patients, reinforcing the role of RNST as a diagnostic rather than severity-grading tool [8,20].

Thymoma was significantly more common in GMG than in OMG (16 vs. 7 patients, *p* < 0.001), reflecting a more-aggressive autoimmune process in GMG, with thymic tumors likely promoting the systemic production of antibodies. This pattern is consistent with a previous report of thymoma being present in 13.6% of GMG patients versus 5.5% of OMG patients [8]. Thymoma-associated MG typically affects older males, while thymic hyperplasia—which is more common in early-onset MG—shows a female predominance. Accordingly, thymectomy was performed more often in GMG patients. Previous studies have demonstrated that thymectomy improves outcomes not only in GMG patients with thymoma but also in GMG patients with thymic hyperplasia or a normal thymus [22, 23]. Notably, the presence of thymoma in seven of the present OMG patients highlights that thymoma can also occur in OMG, indicating the need for chest CT at the initial diagnosis in all MG patients. If this initial screening is negative, routine screening for thymoma at fixed follow-up intervals is not required for well-controlled MG patients. However, repeated CT imaging is warranted if clinical deterioration suggests a missed or developing thymoma.

In our cohort, 20 patients with MG had coexisting autoimmune diseases, most commonly thyroid antibody–related disorders: 5 with hyperthyroidism, 4 with hypothyroidism, and 2 with euthyroid ophthalmopathy. This prevalence appears somewhat higher than that reported previously for European populations (ranging from 10% to 11%) [24,25]. Strabismus due to autoimmune thyroid disease is typically found in 5–10% of MG patients [26]. The presence of ocular motility disorders in both thyroid-associated orbitopathy and MG can make clinical differentiation challenging [27]. In such cases, orbital imaging to assess extraocular muscle enlargement or performing a forced duction test may aid in distinguishing between paralytic strabismus due to MG and restrictive strabismus associated with thyroid-associated ophthalmopathy. Additionally, the incidence of coexisting autoimmune diseases in our cohort was 12.5% in GMG and 15.3% in OMG. Similarly, Guo et al. found that the prevalence of coexisting autoimmune diseases was 21% in GMG and 14% in OMG [5], while Christensen et al. reported rates of 25% and 19%, respectively [22]. These findings collectively underscore the importance of performing assessments for concurrent autoimmune diseases in MG patients through careful history-taking and, when indicated, targeted diagnostic evaluations, regardless of the MG subtype.

In this cohort, treatment patterns differed substantially between MG subtypes, with GMG patients more frequently requiring nonsteroidal immunosuppressive therapy. This likely reflects the broader systemic involvement and higher disease burden characteristic of GMG, necessitating more aggressive immunomodulatory strategies. The preferential use of agents such as tacrolimus and azathioprine in GMG aligns with prior reports emphasizing the need for stronger immunosuppression in patients with generalized disease [1]. The OMG patients with both ptosis and diplopia were more likely to receive systemic immunosuppression than were those with ptosis alone, possibly because the presence of both symptoms indicates a greater disease burden. These findings support the need for tailored treatment strategies based on the symptom profile, disease subtype, and regional response patterns.

Previous studies have consistently demonstrated substantial regional differences in the epidemiology and clinical expression of myasthenia gravis. Western cohorts typically show a bimodal age distribution, with female predominance in early-onset disease and male predominance in late-onset MG, along with higher AChR antibody positivity and more diffuse electrophysiologic involvement, particularly in generalized disease [28–30]. In contrast, Asian cohorts from Taiwan, Japan, and China have been characterized by a higher proportion of early-onset MG, overall female predominance, lower AChR antibody positivity in ocular myasthenia gravis, and a greater tendency toward persistent ocular-limited disease with relatively localized electrophysiologic abnormalities predominantly affecting ocular and facial muscles [9–11,31,32]. Within this context, the present South Korean cohort largely conforms to established Asian patterns, including female predominance, low AChR antibody positivity in OMG, and frequent involvement of the orbicularis oculi muscle on repetitive nerve stimulation testing. At the same time, our cohort demonstrates several distinguishing features, including a particularly marked female predominance in generalized MG, an exceptionally high prevalence of thymoma among GMG patients, and a higher frequency of combined horizontal and vertical diplopia in OMG (Table 5). These observations should be interpreted within the context of a single-center cohort and suggest the need for future multicenter studies to explore genetic, immunologic, and therapeutic factors that may contribute to regional variability in myasthenia gravis [31].

**Table 5:** Comparison of MG characteristics: Western vs. Asian vs. South Korean.

Category	Western Cohorts (US/Europe) ^5,28–30^	East Asian Cohorts (Taiwan/Japan/China) ^9–11,31,32^	South Korean Cohort (Present Study)
Demographics	Bimodal onset; Male dominant in OMG	Early-onset common; Female predominant	Strong female predominance in GMG (70%)
OMG Manifestation	High generalization (50–80%)	Persistent OMG frequent (~50% in China)	Combined H + V diplopia significantly common in OMG
AChR-Ab (OMG)	High (up to 90–95%)	Relatively low (~50%)	Low (36.3%); consistent with Asian traits
RNST Pattern	Distributed across multiple muscles in GMG	Localized to ocular/facial muscles	Orbicularis oculi most affected in both groups
Thymoma (GMG)	Low (~13–14%)	Varies (China ~26.5%, Taiwan ~13%)	High (40%)
Preferred IST	Azathioprine/ Mycophenolate	Azathioprine Tacrolimus use	High Tacrolimus use in GMG (60.0%)

IST: Immunosuppressant Therapy; H + V diplopia: Horizontal and Vertical diplopia

This study had several limitations. Its retrospective design and single-center setting may have reduced the generalizability of the findings. The relatively small samples—particularly within subgroups such as MGFA classifications, thymoma cases, and specific treatment categories—also reduced the statistical power in detecting subtle yet meaningful differences. Large-scale, multicenter prospective studies are therefore warranted to validate these findings.

In conclusion, this study highlights the clinical distinction between ocular and generalized myasthenia gravis within a comparative Western–Asian framework. By characterizing subtype-specific patterns in a South Korean cohort, our findings contribute to the descriptive understanding of regional variability in MG phenotypes. Further multicenter and cross-regional studies will be essential to refine population-specific diagnostic and therapeutic strategies.

## Supporting information

S1 Table Associated autoimmune diseases. (DOCX)
