# Supplementary material for: Comparative study of ocular and generalized myasthenia gravis in a South Korean cohort
Source: PLoS One. 2026 Apr 28;21(4):e0346981. doi: 10.1371/journal.pone.0346981 (PMC13123999; doi:10.1371/journal.pone.0346981)
Supplement: S1 Table — (DOCX) [file pone.0346981.s001.docx]

|  | OMG  (n = 98) | GMG  (n = 40) |
| --- | --- | --- |
| Rheumatoid arthritis | 4 (4.1) | 1 (2.5) |
| Systemic lupus erythematosus | 0 | 0 |
| Hyperthyroidism | 4 (4.1) | 1 (2.5) |
| Hypothyroidism | 2 (2.0) | 2 (5.0) |
| Euthyroid ophthalmopathy | 2 (2.0) | 0 |
| Multiple sclerosis | 0 | 0 |
| Behcet’s disease | 1 (1.0) | 0 |
| Sjogren’s syndrome | 0 | 1 (2.5) |
| Ulcerative colitis | 1 (1.0) | 0 |
| Psoriasis | 1 (1.0) | 0 |
| Total  (patients with ≥1 autoimmune disease) | 15/98 (15.3) | 5/40 (12.5) |

**Supplementary Table 1. Associated autoimmune diseases.**

Data are number (percentage) values.
